# Associations between Oral Hypofunction Tests, Age, and Sex

**DOI:** 10.3390/ijerph181910256

**Published:** 2021-09-29

**Authors:** Yukiko Hatanaka, Junichi Furuya, Yuji Sato, Yoshiki Uchida, Toshiharu Shichita, Noboru Kitagawa, Tokiko Osawa

**Affiliations:** Department of Geriatric Dentistry, School of Dentistry, Showa University, 2-1-1 Kitasenzoku, Ohta-ku, Tokyo 145-8515, Japan; furuyajunichi@gmail.com (J.F.); sato-@dent.showa-u.ac.jp (Y.S.); y.uchida@dent.showa-u.ac.jp (Y.U.); shichita@dent.showa-u.ac.jp (T.S.); kitagawa-n@dent.showa-u.ac.jp (N.K.); tokiko@dent.showa-u.ac.jp (T.O.)

**Keywords:** aging, elderly, oral function, oral frailty, oral hypofunction

## Abstract

Oral function declines in older individuals due to disease and age-related changes, making them vulnerable to oral and physical frailty. Therefore, it is important to manage the decline in oral function in older outpatients. Oral hypofunction is diagnosed by seven tests related to oral function, oral hygiene, oral moisture, occlusal force, oral diadochokinesis, tongue pressure, masticatory function, and swallowing function. However, sex or age were not factored into the current reference values of these tests. We included subjects attending the dental hospital clinic for maintenance, and recorded and analyzed oral hypofunction and the factors associated with its diagnosis. Of the 134 outpatients (53 males and 81 females, mean age 75.2 ± 11.2 years), 63% were diagnosed with oral hypofunction. Oral hypofunction prevalence increased significantly with age, and significant variations were observed in all tests. Furthermore, oral hygiene and swallowing function were not associated with oral hypofunction diagnosis. All examined factors decreased with increasing age, even after adjusting sex, except for oral hygiene and moisture. Occlusal force and masticatory function were higher in men after adjusting age. This study suggested that older outpatients were likely to be diagnosed with oral hypofunction, and that the test reference value and their selection for oral hypofunction should be reconsidered.

## 1. Introduction

Prevention of physical frailty [1] in older people is important to prevent the need for long-term nursing care. A comprehensive approach that includes exercise, sociality, and nutrition has been advocated [2]. Several cross-sectional studies have revealed significant associations between physical frailty and oral frailty in older individuals, suggesting oral frailty, a pathological decline in oral function, might be very important. Watanabe et al. conducted a cross-sectional study of community-dwelling older individuals and reported that oral function was significantly lower in frail participants [3]. Oral frailty in older individuals has also been associated with psychological frailty [4], solitary food consumption [5], and social frailty [6]. 

Oral frailty is characterized by the combined decline in oral functions including tongue, lips, and occlusion [7]. It affects food intake diversity [8], appetite [9], and nutrition intake associated with physical frailty. In a longitudinal study, Tanaka et al. [10] found that a combined decline in oral function including the number of remaining teeth and tongue motor function was associated with lower nutrition, increased risk of frailty, the need for long-term nursing care, and four-year mortality rates in a four year longitudinal study. Therefore, it is important to comprehensively manage oral cavity structures and oral function.

Against this background, the Japanese Society of Geriatric Dentistry defined oral hypofunction in 2016 using seven oral function evaluation criteria [7] to prevent early and accelerated decline of oral function in older people. Oral hypofunction was included in the 2018 Japanese national health insurance system as part of outpatient dental clinical practice that includes the examination and management of oral functions.

Oral hypofunction is diagnosed by performing seven tests to evaluate oral hygiene, oral moisture, occlusal force, tongue–lip motor function, tongue pressure, masticatory function, and swallowing function. Patients are diagnosed with oral hypofunction if three of the seven test scores exceed the reference values [7]. However, the current reference values do not take into consideration sex or age, despite the fact that the oral functions of older individuals are affected by age, sex, and disease [3,11]. Furthermore, large variations in the presence of hypofunction in oral hypofunction tests, and the validity of the reference values have not been sufficiently verified. For example, oral hygiene is more likely to be influenced by lifestyle [12] rather than age, and oral dryness might be influenced by medication [13]. Decline in oral function in older individuals may tend to vary due to individual difference; therefore, it is important to take age and sex into consideration when managing oral function, rather than strictly adhering to uniform reference values. For example, the diagnostic reference value for hypertension and the target value for lowering blood pressure in older individuals are different [14]. 

However, these oral hypofunction problems have only been partially clarified in surveys conducted on community-dwelling older individuals, revealing a high incidence of oral hypofunction [15,16,17,18,19]. The prevalence rates of oral hypofunction in previous studies were about 40–60%, and oral hypofunction was significantly associated with sarcopenia [15], malnutrition [16], frailty [17,18], and mild cognitive impairment [18]. These previous studies have highlighted the importance of oral hypofunction management. Oral hypofunction should be managed in dental outpatient clinics, and dental professionals should provide oral health instruction according to the results of each oral hypofunction test. 

Today, the definition of “older individuals” includes a wide age range as more and more people become even older. It is therefore important to investigate whether oral hypofunction is dependent on age and sex and whether it is a progressive condition. Nevertheless, the association between oral hypofunction tests, age, and sex in dental outpatients have not been fully clarified.

Therefore, this study aimed to conduct a cross-sectional survey and clarify the relationship between the individual test results, age, and sex, and the diagnosis of oral hypofunction in older patients who attended the dental clinic.

## 2. Materials and Methods

### 2.1. Participants

We recruited 134 outpatients (53 males and 81 females, 32–93 years old, mean age 75.2 ± 11.2 years, median age 77.0 years old) visiting a dental clinic at a university dental hospital for oral maintenance that underwent oral function examinations to diagnose oral hypofunction for the first time between September 2018 and July 2020. Written informed consent was obtained from all subjects involved in the study using an opt-out approach. We excluded patients with incomplete data, severe dysphagia, or cognitive decline that made it difficult to perform the examination. This study was approved by the Showa University Ethics Committee (Approval No. DH2018-032).

### 2.2. Data Collection

We extracted basic patient information such as age, sex, and comorbidities, and the test results of oral hygiene, oral dryness, occlusal force, tongue–lip motor function (ODK, oral diadochokinesis), tongue pressure, masticatory function, and swallowing function from the patients’ medical records.

Systemic diseases were scored using the Charlson comorbidity index (CCI) [20]. We also investigated the presence of hypertension, mental diseases other than dementia, degenerative neurological diseases (e.g., Parkinson’s disease), and oral cancer, which are not included in the CCI, but are common in older people and likely to affect oral function. These were considered present if diagnosed by a physician, and if medication had been prescribed within five years for the first three.

Examinations for oral hypofunction were performed as previously described [7]. Oral hygiene was assessed using the tongue coating index (TCI) [21], which involves dividing the tongue surface into nine sections, visually examining each area, and scoring the degree of tongue coating (scores of 0, 1, or 2) to calculate the total score of tongue coating. Oral hygiene was considered hypofunction (poor oral hygiene) if the TCI was >9 (50%).

An oral moisture meter (Mucus, Life Co. Ltd., Saitama, Japan) [22] measured the degree of oral mucosal wetness at the tongue dorsum center. Oral moisture was considered as hypofunction (oral dryness) if the test score was ≤27.9 [7].

The occlusal force of the entire dentition was measured using a pressure-sensitive sheet (Dental Prescale, G.C., Tokyo, Japan) during clenching for three seconds in the occlusal position. The occlusal force was considered hypofunction (decreased occlusal force) if the test score was <500 N [23,24]. Denture wearers were measured with their dentures in place. The number of remaining teeth, used as an alternative examination method, was calculated after excluding remaining tooth roots and teeth with vertical movement due to periodontal disease [7].

The tongue–lip motor function was evaluated using ODK [25]. An automatic measuring device (Kenkuchi-kun Handy, Takei Kikai Kogyo, Niigata, Japan) was used to measure the number of syllables pronounced per second by having subjects pronounce each monosyllable (/pa/, /ta/, /ka/) for five seconds. The tongue–lip motor function was considered hypofunction (decreased tongue–lip motor function) if the test score was <6 per second [7].

Tongue maximum pressure was measured using a tongue depressor (JMS Tongue Depressor, G.C., Tokyo, Japan). The tongue pressure was considered as hypofunction (decreased tongue pressure) if the test score was <30 kPa [7].

The masticatory function was evaluated based on the amount of glucose eluted when freely chewing 2 g of gummy jelly (Glucoram, G.C., Tokyo, Japan) for 20 s. The amount of glucose elution was measured using a masticatory ability test system (Glucosensor GS-II, G.C., Tokyo, Japan). The masticatory function was considered hypofunction (decreased masticatory function) if the glucose concentration was <100 mg/dL [7].

Swallowing function was assessed using the subjective swallowing screening questionnaire (The 10-item Eating Assessment Tool, EAT-10). Swallowing function was considered hypofunction (decreased swallowing function) if the test score was ≥3 [7].

Oral hypofunction was diagnosed when at least three of seven tests were considered hypofunction [7].

### 2.3. Statistical Analysis

We analyzed the data for an association between oral hypofunction prevalence, age, and sex (χ^2^ test), the rate of hypofunction of each examination (Q test), factors related to oral hypofunction diagnosis (logistic regression analysis), a correlation between oral function test values and age (Spearman’s correlation coefficient), and the relationship between the oral function test values and age, sex, and comorbidities (multiple regression analysis). IBM SPSS Statistics for Windows, Version 27.0 (IBM Corp., Armonk, NY, USA) was used for statistical analysis, and the significance level was set at 5%.

## 3. Results

### 3.1. Patient Characteristics

**Figure 1:**
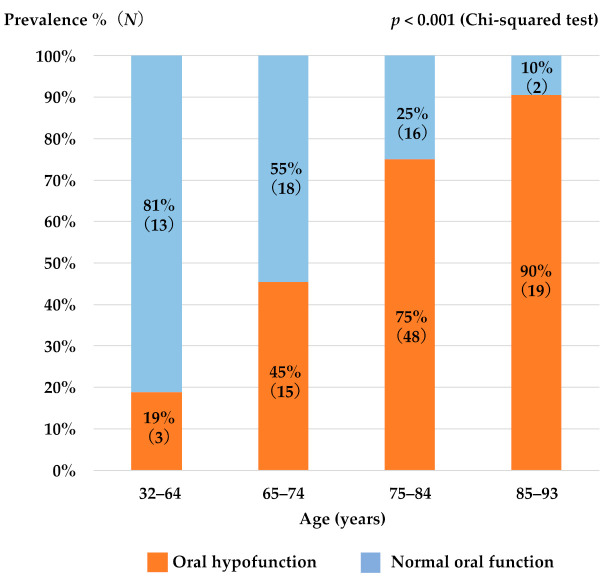
Oral hypofunction prevalence and age. Oral hypofunction prevalence increased significantly with age zone (*p* < 0.001, Chi-squared test).

**Figure 2:**
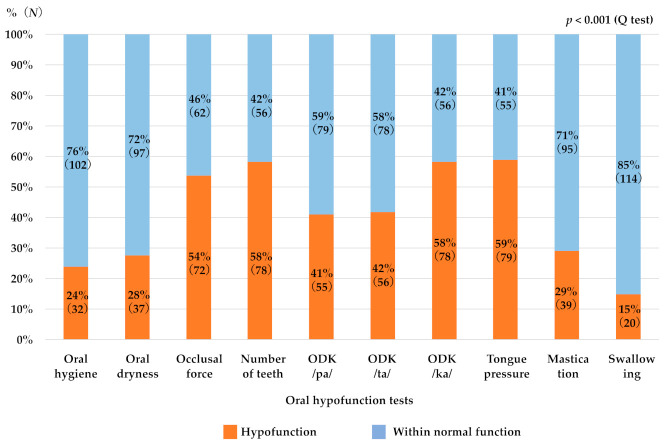
The rate of hypofunction diagnosed with the current reference value in oral hypofunction tests. The rate of hypofunction varied significantly between tests (*p* < 0.001, Q test).

Table 1 shows the basic patient information, oral hypofunction prevalence, and test values for all oral examinations. Of the 134 subjects, 63% had oral hypofunction. The male (66%) and female (62%) rates were similar. The oral hypofunction prevalence (the ratio of patients who were diagnosed as oral hypofunction) increased significantly with age, affecting approximately 75% of those aged 75–84 years, and 90% of those aged 85 years and older (Figure 1). The rates of hypofunction diagnosed with current reference values in each oral test varied significantly (Figure 2). The rate of hypofunction was approximately 20% for oral hygiene, oral dryness, masticatory function, and swallowing function, and approximately 50% for occlusal force, number of teeth, ODK, and tongue pressure.

### 3.2. Factors Associated with Susceptibility to Oral Hypofunction

Table 2 shows the logistic regression analysis results using oral hypofunction diagnosis as the objective variable and age, sex, CCI, presence of hypertension, and the seven test items as explanatory variables. Mental disorders, degenerative neurological diseases, and oral cancer were excluded from the analysis due to the small number of participants in each and the risk of multicollinearity. We found an association between oral hypofunction diagnosis and older age, oral dryness, and decreased occlusal force, decreased tongue–lip motor function, decreased tongue pressure, and decreased masticatory function, while poor oral hygiene and decreased swallowing function were not.

### 3.3. Correlation between Each of the Oral Hypofunction Test Item and Age

The correlations between the results of each oral hypofunction test and age are presented in Table 3. Poor oral hygiene and oral dryness were not correlated with age. We observed weak or moderate negative correlations between age and occlusal force, the number of remaining teeth, tongue pressure, ODK (/pa/, /ta/, /ka/), and masticatory function. There was a weak positive correlation between EAT-10 values and age (i.e., a correlation between age and decreased swallowing function). When analyzed by sex, weak negative correlations were found between age and the number of teeth, ODK (/pa/, /ka/), and tongue pressure in males, and weak or moderate correlations with all items except oral hygiene and oral dryness in females. Therefore, the sexes differed in how age correlated with each of the oral hypofunction tests.

### 3.4. Relationship between Each Oral Hypofunction Test Item and Age, Sex, and Comorbidities

Table 4 shows the multiple regression analysis results with the oral function tests as the objective variables and age, sex, CCI, and hypertension as the explanatory variables.

Oral hygiene and oral moisture were not associated with age, sex, or comorbidities, similar to the univariate analysis results. Occlusal force and masticatory function showed a significant negative association with age and sex independently. The number of teeth, ODK, tongue pressure, and swallowing function were significantly negatively associated with age but not with sex or comorbidities.

## 4. Discussion

In the present study, large variations in the presence of hypofunction in each oral test were observed, suggesting that the current reference values for the diagnosis of oral hypofunction should be reconsidered. Furthermore, our findings show that occlusal force caused by teeth clenching and masticatory function caused by chewing a gummy jelly were independently related to sex and age, while oral hygiene and oral moisture were not. Therefore, age and sex should be considered when determining the reference value for the diagnosis and management of oral hypofunction.

The participants in this study were independent patients attending the dental outpatient clinic. Although they were relatively old, they had few diseases, other than hypertension, that could affect their oral function and were considered robust older adults. It has been reported that the oral hypofunction test results could be easily influenced by dental complaints [19]. However, dental complaints vary greatly among patients and are difficult to analyze. Therefore, in this study, we included patients attending the hospital for maintenance, rather than for treatment.

Previous studies on older individuals living in the community, rather than outpatients, reported an oral hypofunction prevalence of about 50–60% [15,17,18] and 43% [16]. The oral hypofunction prevalence reported in this study was higher (63%). This was thought to be due to the fact that the participants in our study were all outpatients visiting a dental hospital. However, the rate was only 36% in a previous study that included dental hospital outpatients [19]. This could be attributed to the older participants in our study. In general, older patients who attend dental clinics for maintenance may have better oral health literacy and oral function than average population. Similarly, participants in previous studies might have good health literacy and oral function because they were evaluated at on-site health check-up. Therefore, further large-scale cross-sectional studies considering the relationship between oral health literacy, gerotranscendence [26], and oral health will be needed in the future.

The oral hypofunction prevalence in patients aged 75 and older was over 75% and the prevalence in patients aged 85 years and older was over 90%. Although the decline in oral function could undoubtedly be caused by age-related changes, a very high percentage of older people might fall into the category of oral hypofunction determined by uniform reference values that do not factor for age.

Furthermore, the multiple analysis revealed that oral hygiene and swallowing function were unlikely to be associated with oral hypofunction diagnosis. Among the seven tests used to diagnose oral hypofunction, oral hygiene is diagnosed based on tongue coat, which might be more related to lifestyle factors such as oral cleaning and diseases than age. A study of stroke patients showed that tongue hygiene was poorly associated with tongue motor function [11]; therefore, although rarely, oral hygiene could also be related to oral functions such as tongue function. The EAT-10 questionnaire used to evaluate swallowing function is a subjective evaluation tool that might indicate the need for a detailed examination for swallowing disorders. Therefore, further research is needed to determine whether the seven tests are valid diagnostic items for oral hypofunction according to its aging- or disease-related progression.

The oral tests were associated with different levels of diagnosis of oral hypofunction because of the variations in the presence of hypofunction with current reference values. The rates of hypofunction were approximately 20% for oral hygiene, oral dryness, masticatory function, and swallowing function, and approximately 50% for occlusal force, number of teeth, ODK, and tongue pressure. This trend resembled the results of the study by Kugimiya et al. [15], while there was a large difference in the presence of hypofunction at each oral test compared to the study by Shimazaki et al. [17]. Since oral hypofunction is diagnosed based on three of the seven tests, the fact that the results of the oral tests tended to differ depending on the study is problematic and suggests the need to revalidate the criteria to diagnose oral hypofunction.

We used multiple analysis, adjusted for sex and comorbidities to investigate the correlation between each test item and age. The findings showed that oral hygiene and oral moisture were not associated with age, while other oral functions were negatively correlated with age, even after adjusting for sex and comorbidities. Kugimiya et al. [15] evaluated the median value of each oral test in each age group, showing that the values decreased with the increase in age in all items other than oral hygiene and dryness. Oral hygiene and moisture were considered weakly related to age. This could be because they are influenced by oral hygiene habits, drinking habits, and medications, rather than by age. Occlusal force and masticatory function were higher in males than in females, even after adjusting for age while the number of teeth was not affected by sex, suggesting that the sex difference in muscle strength between males and females affected the values of occlusal force and masticatory function. Similarly, it was assumed that sex differences in tongue pressure could occur. However, Utanohara et al. [27] reported that sex differences were less likely to occur after the age of 50, which supports the results of this study.

Managing oral function in older people in dental outpatient clinics is an important way to prevent frailty [28], which might help prevent malnutrition [16]. However, the findings of this study revealed the necessity of revalidating the current reference values, the appropriateness of using three of seven for diagnosis, and the necessity of weighting each test and setting target values while considering age and sex to manage oral hypofunction properly.

This study was a cross-sectional survey. Therefore, one of its limitations is that we were unable to determine causal relationships. Longitudinal and interventional studies with patients diagnosed with oral hypofunction are needed. Since this study was conducted at a single institution on robust patients attending the clinic for maintenance, their oral functions might differ from those of patients visiting the clinic with dental complaints or severe comorbidities.

## 5. Conclusions

This study showed that the older the dental outpatients, the more likely they were to be diagnosed with oral hypofunction; however, large variations of hypofunction were noted in the oral hypofunction tests. Oral hygiene and swallowing function were poorly associated with oral hypofunction diagnosis. All oral function tests other than oral hygiene and oral moisture showed a negative relationship with age. Occlusal force and masticatory function were higher in men than women due to sex difference. Therefore, the tests’ reference values and their selection for oral hypofunction should take into consideration age and sex when diagnosing and managing patients attending dental outpatient clinics.

## Figures and Tables

**Table 1:** Patient characteristics.

Measurement Items	Mean ± SD			
	Number (%)			
	Overall (*n* = 134)	Male (*n* = 53)	Female (*n* = 81)	*p*-Value
Age *	75.2 ± 11.2	77.6 ± 9.2	73.6 ± 12.2	0.034
CCI	0.5 ± 0.8	0.7 ± 1.0	0.4 ± 0.7	0.077
Hypertension	50 (37.3)	25 (18.7)	25 (18.7)	0.056
Mental disorder	7 (5.2)	3 (2.2)	4 (3.0)	0.854
Neurodegenerative disease	1 (0.02)	1 (0.02)	0 (0.0)	0.215
Oral cancer	0 (0.0)	0 (0.0)	0 (0.0)	n/a
Oral hypofunction	85 (63.4)	35 (66.0)	50 (61.7)	0.613
Oral hygiene	28.3 ± 21.7	26.1 ± 22.2	29.6 ± 21.0	0.304
Oral dryness	28.0 ± 3.2	28.6 ± 3.4	27.7 ± 3.0	0.091
Occlusal force	563.4 ± 391.5	645.0 ± 398.4	510.0 ± 379.9	0.039
Number of remaining teeth	15.3 ± 9.2	15.1 ± 9.0	15.4 ± 9.4	0.748
ODK /pa/ *	5.9 ± 1.1	5.7 ± 1.0	6.0 ± 1.1	0.028 *
ODK /ta/	6.0 ± 1.0	5.9 ± 0.9	6.1 ± 1.1	0.132
ODK /ka/ *	5.6 ± 1.0	5.4 ± 0.9	5.7 ± 1.0	0.046 *
Tongue pressure	26.9 ± 8.6	27.9 ± 8.5	26.2 ± 8.7	0.290
Masticatory function	132.6 ± 58.9	143.6 ± 62.1	125.4 ± 55.9	0.087
Swallowing function	1.6 ± 4.0	1.3 ± 3.7	1.8 ± 4.2	0.207

* *p* < 0.05, Male vs. Female, Mann–Whitney *U* test or Chi-squared test. SD, standard deviation; CCI, Charlson’s comorbidity index; ODK, oral diadochokinesis; n/a, not applicable.

**Table 2:** Logistic regression analysis with oral hypofunction as the objective variable.

Explanatory Variables	Odds Ratio	95% CI	*p*-Value
Age *	1.076	1.006–1.151	0.033 *
Sex (0 = male, 1 = female)	0.310	0.075–1.285	0.107
CCI	0.682	0.280–1.661	0.400
Hypertension (1 = positive)	1.548	0.422–5.677	0.510
Oral hygiene	1.154	0.960–1.386	0.127
Oral dryness *	0.636	0.475–0.852	0.002 *
Occlusal force *	0.995	0.993–0.998	<0.001 *
ODK lowest *	0.221	0.085–0.577	0.002 *
Tongue pressure *	0.890	0.796–0.994	0.038 *
Masticatory function *	0.982	0.969–0.995	0.008 *
Swallowing function	1.298	0.886–1.903	0.180

* *p* < 0.05, Logistic regression analysis. Objective variable is the diagnosis of oral hypofunction (Normal oral function = 0, Oral hypofunction = 1). All explanatory variables other than sex and hyper tension were continuous variables. ODK lowest: the lowest among the oral diadochokinesis values of /pa/, /ta/, and /ka/. ODK, oral diadochokinesis; CCI, Charlson’s comorbidity index; CI, confidence interval.

**Table 3:** Correlation analysis between oral hypofunction tests and age.

Oral Hypofunction Tests	All (*n* = 134)		Male (*n* = 53)		Female (*n* = 81)	
	r	*p*-Value	r	*p*-Value	r	*p*-Value
Oral hygiene	−0.074	0.395	−0.049	0.729	−0.061	0.590
Oral dryness	0.104	0.232	0.117	0.403	0.031	0.786
Occlusal force	−0.239	0.005 *	−0.199	0.154	−0.305	0.006 *
Number of remaining teeth	−0.407	<0.001 *	−0.301	0.029 *	−0.446	<0.001 *
ODK /pa/	−0.389	<0.001 *	−0.318	0.020 *	−0.392	<0.001 *
ODK /ta/	−0.324	<0.001 *	−0.232	0.094	−0.345	0.002 *
ODK /ka/	−0.426	<0.001 *	−0.357	0.009 *	−0.454	<0.001 *
Tongue pressure	−0.274	0.001 *	−0.413	0.002 *	−0.226	0.042 *
Masticatory function	−0.238	0.006 *	−0.184	0.186	−0.316	0.004 *
Swallowing function	0.289	0.001 *	0.24	0.083	0.346	0.002 *

* *p* < 0.05, Spearman’s rank correlation coefficient; ODK, oral diadochokinesis.

**Table 4:** Multiple regression analysis with oral hypofunction tests as the objective variable.

Oral Hypofunction Tests	Explanatory Variables	B	SE	β	*p*-Value	95% CI	Variance Inflation Factor
Oral hygiene	Age	−0.006	0.032	−0.018	0.850	−0.069 to 0.057	1.113
	Sex	0.595	0.709	0.076	0.403	−0.807 to 1.998	1.070
	CCI	−0.299	0.432	−0.062	0.491	−1.154 to 0.557	1.062
	HT	0.662	0.722	0.084	0.360	−0.765 to 2.090	1.085
Oral moisture	Age	0.005	0.026	0.019	0.837	−0.046 to 0.056	1.113
	Sex	−0.885	0.579	−0.138	0.129	−2.031 to 0.260	1.070
	CCI	−0.014	0.353	−0.003	0.969	−0.712 to 0.685	1.062
	HT	0.198	0.589	0.030	0.738	−0.968 to 1.364	1.085
Occlusal force	Age *	−9.031	3.044	−0.259	0.004 *	−15.055 to −3.008	1.113
	Sex *	−175.828	68.258	−0.220	0.011 *	−310.879 to −40.777	1.070
	CCI	−63.037	41.636	−0.129	0.132	−145.415 to 19.342	1.062
	HT	79.469	69.478	0.099	0.255	−57.994 to 216.932	1.085
Number of remaining teeth	Age *	−0.345	0.069	−0.420	<0.001 *	−0.482 to −0.208	1.113
	Sex	−1.025	1.553	−0.055	0.510	−4.097 to 2.047	1.070
	CCI	−0.727	0.947	−0.063	0.444	−2.601 to 1.147	1.062
	HT	1.029	1.580	0.054	0.516	−2.098 to 4.155	1.085
ODK/pa/	Age *	−0.033	0.008	−0.346	<0.001 *	−0.049 to −0.017	1.113
	Sex	0.151	0.181	0.070	0.405	−0.207 to 0.510	1.070
	CCI	−0.126	0.111	−0.095	0.257	−0.345 to 0.093	1.062
	HT	0.011	0.184	0.005	0.954	−0.354 to 0.376	1.085
ODK/ta/	Age *	−0.028	0.008	−0.302	0.001 *	−0.043 to −0.012	1.113
	Sex	0.010	0.175	0.005	0.957	−0.337 to 0.356	1.070
	CCI	−0.175	0.107	−0.136	0.104	−0.386 to 0.037	1.062
	HT	−0.227	0.178	−0.107	0.204	−0.580 to 0.125	1.085
ODK/ka/	Age *	−0.030	0.007	−0.345	<0.001 *	−0.045 to −0.015	1.113
	Sex	0.122	0.167	0.061	0.466	−0.208 to 0.451	1.070
	CCI	−0.090	0.102	−0.074	0.377	−0.291 to 0.111	1.062
	HT	−0.067	0.170	−0.034	0.692	−0.403 to 0.268	1.085
Tongue pressure	Age *	−0.171	0.069	−0.223	0.014 *	−0.307 to −0.036	1.113
	Sex	−2.745	1.538	−0.156	0.077	−5.787 to 0.297	1.070
	CCI	−0.684	0.938	−0.064	0.467	−2.539 to 1.172	1.062
	HT	−0.868	1.565	−0.049	0.580	−3.965 to 2.228	1.085
Masticatory function	Age *	−1.172	0.461	−0.223	0.012 *	−2.084 to −0.260	1.113
	Sex *	−25.522	10.335	−0.213	0.015 *	−45.969 to −5.075	1.070
	CCI	−12.194	6.304	−0.166	0.055	−24.666 to 0.279	1.062
	HT	4.578	10.519	0.038	0.664	−16.235 to 25.390	1.085
Swallowing function	Age *	0.100	0.032	0.280	0.002*	0.037 to 0.164	1.113
	Sex	0.738	0.718	0.090	0.306	−0.683 to 2.159	1.070
	CCI	−0.034	0.438	−0.007	0.939	−0.900 to 0.833	1.062
	HT	−0.944	0.731	−0.114	0.199	−2.391 to 0.502	1.085

* *p* < 0.05, multiple regression analysis. All independent variables were continuous variables. CCI, Charlson’s comorbidity index; HT, hypertension (0 = absence, 1 = presence); ODK, oral diadochokinesis; CI, confidence interval.

## Data Availability

Not applicable.
